# Synthesis of Bis(isodecyl Terephthalate) from Waste Poly(ethylene Terephthalate) Catalyzed by Lewis Acid Catalysts

**DOI:** 10.3390/ijms252312953

**Published:** 2024-12-02

**Authors:** Marcin Muszyński, Janusz Nowicki, Agata Krasuska, Ewa Nowakowska-Bogdan, Maria Bartoszewicz, Piotr Woszczyński, Mateusz Zygadło, Gabriela Dudek

**Affiliations:** 1 Department of Physical Chemistry and Technology of Polymers, Faculty of Chemistry, PhD School, Silesian University of Technology, ks. M. Strzody 9, 44-100 Gliwice, Poland; 2 Łukasiewicz Research Network—Institute of Heavy Organic Synthesis “Blachownia”, Energetyków 9, 47-225 Kędzierzyn-Koźle, Poland; janusz.nowicki@icso.lukasiewicz.gov.pl (J.N.); agata.krasuska@icso.lukasiewicz.gov.pl (A.K.); ewa.nowakowska@icso.lukasiewicz.gov.pl (E.N.-B.); maria.bartoszewicz@icso.lukasiewicz.gov.pl (M.B.); piotr.woszczynski@icso.lukasiewicz.gov.pl (P.W.); 3 Department of Physical Chemistry and Technology of Polymers, Faculty of Chemistry, Silesian University of Technology, Chemistry Students Research Society ks. M. Strzody 9, 44-100 Gliwice, Poland; matezyg797@student.polsl.pl; 4 Department of Physical Chemistry and Technology of Polymers, Faculty of Chemistry, Silesian University of Technology, ks. M. Strzody 9, 44-100 Gliwice, Poland

**Keywords:** recycling, PET, alcoholysis

## Abstract

Increasing plastic waste generation has become a pressing environmental problem. One of the most produced waste plastics originates from post-consumer packaging, of which PET constitutes a significant portion. Despite increasing recycling rates, its accumulation has created a need for the development of new recycling methods that can further expand the possibilities of recycling. In this paper, we present the application of Lewis acid catalysts for the depolymerization of PET waste. The obtained results show the formation of diisodecyl terephthalate (DIDTP), which is used as a PVC plasticizer. For this purpose, several Lewis acid catalysts were tested, including tin, cobalt, manganese, zirconium, zinc, and calcium derivatives, alongside zinc acetate and potassium hydroxide, which were used as reference catalysts. Our results show that tin (II) oxalate is the most effective catalyst, and it was then used to synthesize two application samples (crude and purified). The physicochemical properties of PVC mixtures with the obtained samples were determined and compared to commercial plasticizers, where both plasticizers had similar plasticizing properties to PVC plasticization.

## 1. Introduction

Plastics are a group one of the most widely produced chemicals globally, reaching a worldwide production level of 359 million tons, which increased in 2020 by approximately 8 million tons. A large portion of these constitutes poly(ethylene terephthalate) (PET), whose production reached 35 million tons [1]. The ever-increasing plastic production due to increasing demand and poor waste management has led to increased plastic waste generation and accumulation in the environment. Since many types of plastic tend to accumulate in the environment in the form of microplastics, which tends to pollute marine life, the problem of recycling has become more severe in recent years [2,3] due to plastics’ poor biodegradability [4,5]. 

The increased generation of PET waste and the need for its utilization resulted in the development of numerous recycling methods [6,7,8]. PET recycling can be divided into the following main branches: material, thermochemical (pyrolysis) [9,10], and chemical recycling. 

Waste PET can be recycled using material recycling. Using this method, PET packaging can be reused by sorting and subsequent purification and remelting. Waste PET can also find applications as additives for different types of materials. Chemically recycled PET can be used in paving materials as an additive [11] or after undergoing chemical recycling, for example, the aminolysis process. The obtained additive has a positive effect on asphalt mixture properties [12,13,14]. 

Thermochemical recycling relies on treating PET wastes using high temperatures reaching 450 °C and elevated pressure in an oxygen-free environment. The resulting liquid product consists of oligomeric polymer fragments and polymer building blocks, like terephthalic acid (TPA) and ethylene glycol [15]. Pyrolysis can also be used to obtain fuel fractions [16]. The thermochemical recycling method has several drawbacks. Most notably, it results in the degradation of monomers, and especially in the case of fuel production, the monomers are not reclaimed and cannot be reused for polymer synthesis. Therefore, other methods of chemical recycling of PET are more beneficial. Among these methods, which are considered to be more environmentally friendly, are hydrolysis, glycolysis, and alcoholysis [6,17,18,19,20,21]. 

The hydrolysis of PET uses water as a reactant to break ester bonds in polymers, producing, as final products terephthalic acid and ethylene glycol. Hydrolysis can be conducted in acid, alkaline, and neutral conditions [18,22]. Alkaline hydrolysis allows achieving high yields of 99% of terephthalic acid [23,24,25,26,27,28]; however, the initial product of these reactions are terephthalic acid salts, which must be decomposed to terephthalic acid. This is usually achieved by acids with the use of strong acids, like H_2_SO_4_, which is disadvantageous for the process economy due to the introduction of additional process steps and the increased usage of materials. Acid-catalyzed hydrolysis is conducted without the additional decomposition of terephthalic acid salts, generating fewer byproducts. The reaction is performed using such catalysts as sulfuric acid [29], p-toluene sulfonic acid [29], heterpolyacids [29], and superacids [30,31]. Acidic PET hydrolysis can also be catalyzed by terephthalic acid, which is one of the main products of the reaction [32]. The reaction can also be conducted without the use of a catalyst. In this case, supercritical and subcritical reaction conditions are usually implemented. Such reaction often results in high conversion rates [26,33]. 

Glycolysis is a widely used method of the chemical recycling of poly(ethylene terephthalate). It incorporates the use of glycols, most commonly ethylene glycol [18], as a reactive solvent or in the presence of a cosolvent [34]. The reaction is carried out to obtain bis(hydroxyethyl) terephthalate (BHET) as a main product or PET oligomers, which can be used in PET synthesis. A number of different catalysts have been used in this process, most notably alkali metal acetates, such as zinc, manganese, cobalt, and led acetates [35,36,37]; acetate complexes [38]; inorganic salts [39,40]; metal oxides [41,42,43,44,45], polyoxymetalates [46]; and ionic liquids [47,48,49].

Alcoholysis using monohydric alcohols is applied in the synthesis of terephthalic esters using various types of alcohol, including methanol [50,51,52,53,54,55,56], ethanol [57,58,59,60,61], butanol [62], pentanol [63], and higher alcohols, such as 2-ethylhexanol [64,65,66,67,68] and isononyl and decyl alcohols [69]. The alcoholysis process can be conducted with or without a catalyst. Goto et al. [70] carried out the synthesis of dimethyl terephthalate from PET using a noncatalytic process and in the presence of zinc acetate as a catalyst. The reaction yield obtained without the use of a catalyst reached 80% wt. at 350 °C. Incorporating an acetate catalyst had a beneficial effect in increasing the reaction yield at the lower temperature of 270 °C. Acetate is considered to be an efficient PET alcoholysis catalyst, allowing to obtain high reaction yields [51,54,57]. Among other catalytic systems, a range of catalysts are used, like inorganic salts, alkoxides [71], ionic liquids [72,73], organic bases [74], and organometallic compounds [67]. The process can also be conducted under supercritical conditions [60,61]. The alcoholysis of waste PET using higher alcohols ranging from octanol to decanol is of particular importance, since the obtained products can be used as plasticizers. 

Liu et al. used propyltriethylammonium chloroironinate ([HO_3_S–(CH_2_)_3_–NEt_3_]Cl–FeCl_3_) as acosolvent [66] in PET alcoholysis with 2-ethylhexanol. Various molar fractions of zinc, copper, and iron chlorides were tested as reaction catalysts. It was found that the addition of the FeCl_3_ molar fraction between 0.67 and 0.75 at the temperature of 210 °C with a 8h reaction time using 3.39 excess of 2-ethylhexanol resulted in 100% conversion of PET and DOTP, with a yield of over 97%. Tetrabutyl titanate and zinc acetate were tested during dioctyl terephthalate (DOTP) synthesis and investigated as plasticizers by Chen et al. [67]. The process was conducted using ionic liquids as cosolvents. It was found that the addition of titanium butoxide to butylmethylimidasolium chloride had a beneficial effect on the reaction yield, increasing it from 43 to 86%. The application of titanium butoxide and zinc chloride as reference catalysts resulted in lower yields than the tested compounds. 

Deep eutectic solvents (DESs), like choline chloride, are also used in PET alcoholysis. Zhou et al. [68] investigated the use of DES comprising choline chloride and metal salts, like zinc, manganese, cobalt, and copper acetates, as well as FeCl_3_, CoCl_2_, tetrabutyl titanate, and sulfuric acid in PET depolymerization with 2-ethylhexanol. The activity of DESs was compared to those of other transesterification catalysts. The applied deep eutectic solvents demonstrated a high activity in PET depolymerization, which resulted in conversion rates reaching up to 100% for ChCl/Zn(Ac)2 and a DOTP yield of 84.2%. 

Organic metal compounds are active catalysts in the esterification reaction to prepare trimethylene terephthalate using tetrabutyl titanate and dibutyltin oxide [75] in transesterification reaction [76], polyester synthesis [77], and the depolymerization of polymers [78]. Waste PET alcoholysis can be conducted using monohydric alcohols, such as 2-ethylhexanol [64,65,79] and isononyl and isodecyl alcohols [69], to produce plasticizers. Poller et al. [80] studied a series of alkyl and aryltin compounds, e.g., dimethyltin oxide, dibutyltin oxide, dibutyltin chloride, dibutyltin diacetate, diphenyltin chloride, triphenyltin acetate, and others, in the transesterification of *n*-propyl acetate with methanol. The authors found that dibutyltin acetate and dibutyltin oxide exhibited the highest catalytic activity. One of the earliest studies conducted on PET depolymerization to produce plasticizers was conducted by Dupont et al. [64,65]. The authors conducted alcoholysis trials using different types of PET and PBT from various sources, such as beverage bottles, food packaging and packaging films, fabrics, and car parts, and X-ray and photographic films, with tin oxalate as a catalyst, were used. The obtained products were then tested for their PVC plasticization properties. The physicochemical properties of the PVC-plasticizer mixture, such as tensile strength, elongation at break, hardness, and brittle point, were very similar. One notable difference was that the color of the plasticizer and its mixture with PVC was heavily influenced by the color of PET applied in synthesis. The incorporation of tin oxalate resulted in good reaction yields; however, a notable increase in the DOTP concentration occurred after three hours of the reaction. The application of organotin catalysts in the synthesis of DOTP from waste poly(ethylene terephthalate) was conducted by Muszyński et al. [81]. In this study, the authors investigated the alcoholysis of PET waste with 2-ethylhexanol in the presence of various organotin compounds, with zinc acetate and potassium hydroxide as the reference catalysts. The applied tin catalysts showed a good activity, resulting in high DOTP yields ranging from 85 to 99% for tin (II) bis(2-ethylhexanoate) and monobutyltin *tris*(2-ethylhexanoate), respectively. Higher alcohols, including 2-ethylhexanol, isononyl alcohol, and decyl and benzyl alcohols, as well as glycerol, were used in the depolymerization of waste PET with tetrabutyl titanate as a catalyst to produce rubber plasticizers [69]. The application of these catalysts resulted in high terephthalate yields, reaching 98% for glycerol and 96%, 96,91%, and 89% for benzyl, isononyl, and decyl alcohols, respectively. 

**Figure 1:**
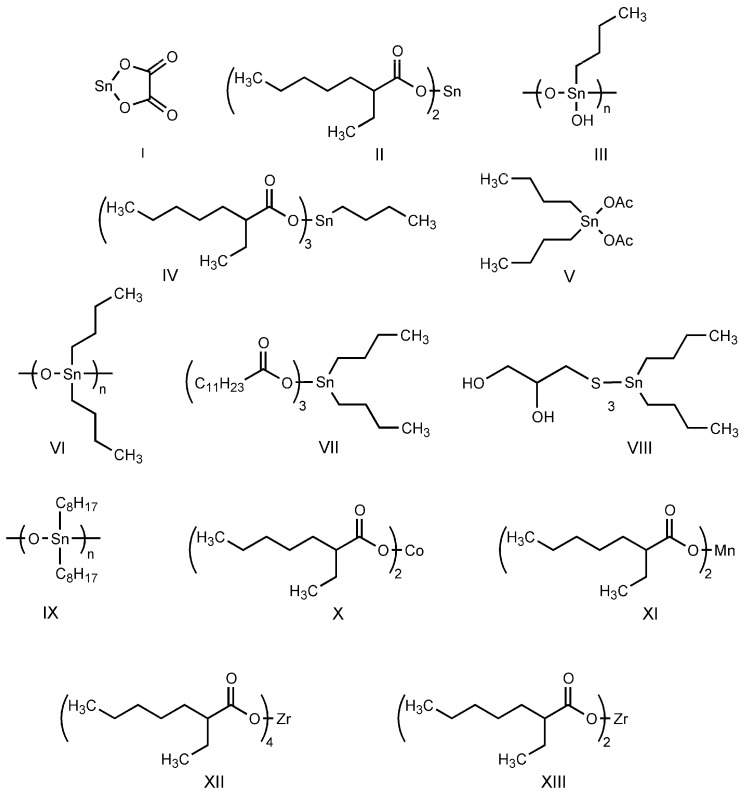
Structures of the applied catalysts.

In this study, we examine the application of previously untested Lewis acid catalysts in poly(ethylene terephthalate) depolymerization using PET alcoholysis in the presence of isodecyl alcohol. Several Lewis acid catalysts were tested, including tin (II) oxalate (**I**), tin (II) bis(2-ethylhexanoate) (**II**), monobutyltin oxide (**III**), monobutyltin dihydroxychloride (**IV**), monobutyltin tris(2-ethylhexanoate) (**V**), dibutyltin diacetate (**VI**), dibutyltin oxide (**VII**), dibutyltin dilaurate (**VIII**), dibutyltin bis(1-thioglyceride) (**IX**), dioctyltin oxide, cobalt (II) 2-ethylhexanoate (**XI**), manganese (II) 2-ethylhexanoate (**XII**), zirconium (IV) 2-ethylhexanoate (**XIII**), zinc (II) 2-ethylhexanoate (**XIV**), cobalt (II) neodecanate (**XV**), zinc (II) neodecanate (**XVI**), and calcium (II) 3,5,5-trimethylhexanoate (**XVII**) (Figure 1). Metallo-organic compounds proved to be effective transesterification and esterification catalysts. 

We predict that incorporating such catalysts in PET depolymerization in the presence of isodecyl alcohol will allow for effective alcoholysis to obtain diisodecyl terephthalate (DIDTP) with a good yield and a relatively low byproduct concentration. These catalysts showed a greater catalytic activity compared to the standard reference catalysts, like zinc acetate, which, as was shown in previous research, exhibits a relatively low activity during the first hour of the reaction. The reaction conducted with these new catalysts is also expected to yield lower concentrations of byproducts than inorganic hydroxides, ensuring greater reaction yields. A catalyst screening procedure was implemented to select the most effective catalyst. Purified and unpurified products were obtained, and their plasticization properties were tested in PVC mixtures. The physicochemical properties were measured and compared to those of commercial-grade plasticizers.

## 2. Results and Discussion

### 2.1. Catalyst Screening

The alcoholysis of waste PET was conducted using the catalysts shown in Figure 1. Zinc acetate and potassium hydroxide were also applied as reference catalysts. They are widely used as alcoholysis catalysts and various other reactions, and can be used to compare the catalytic performance of the tested substances. Zinc acetate has shown a good catalytic activity in the alcoholysis reaction, which resulted in a DIDTP concentration of 33.7% wt. in the reaction mixture after six hours. However, the initial concentration of DIDTP was low, and after 1 h of the process, only 12.4% wt. of DIDTP was present in the reaction mixture, and an amount of unreacted PET was observed in the reactor. The DIDTP concentration increased to 25.6% wt. after three hours. All of the PET was dissolved after four hours. After six hours, the DIDTP concentration reached 33.7% wt., and the yield was 81.5%. The depolymerization conducted using potassium hydroxide resulted in a post-reaction mixture with approximately 35.0% wt. of post-reaction mixture mass consisting of a solid phase, which resulted in a low DIDTP concentration of 19.2% wt. and a high amount of byproducts, reaching 45.3% wt. 

**Figure 2:**
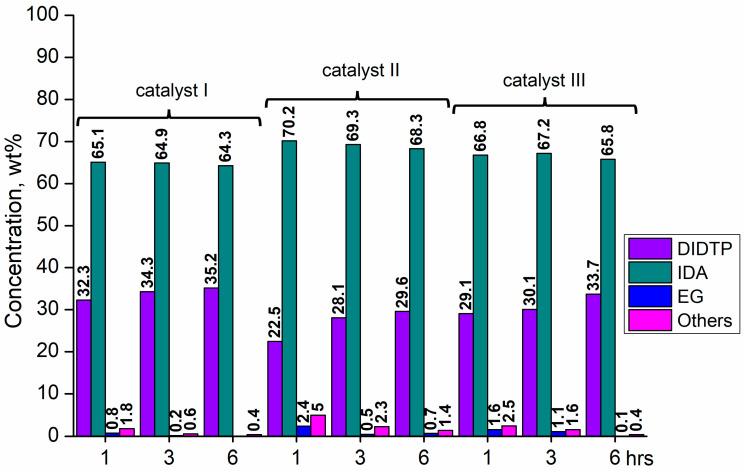
The reaction mixture composition during the depolymerization of PET waste using: tin (II) oxalate (**I**), tin (II) bis(2-ethylhexanoate) (**II**), and monobutyltin oxide (**III**).

**Figure 3:**
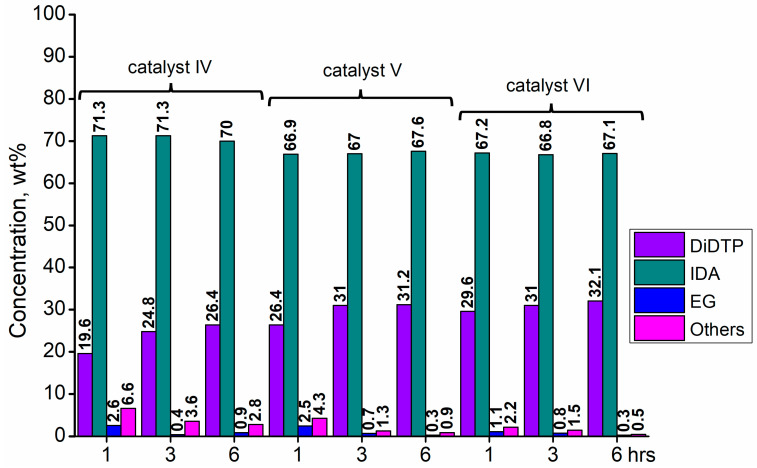
The reaction mixture composition during the depolymerization of PET waste using: monobutyltin dihydroxychloride (**IV**), monobutyltin tris(2-ethylhexanoate) (**V**), and dibutyltin diacetate (**VI**).

**Figure 4:**
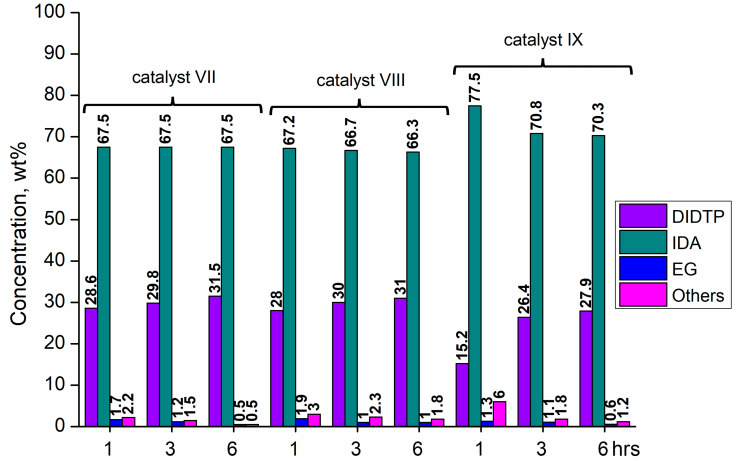
The reaction mixture composition during the depolymerization of PET waste using: dibutyltin oxide (**VII**), dibutyltin dilaurate (**VIII**), and dibutyltin bis(1-thioglyceride) (**IX**).

**Figure 5:**
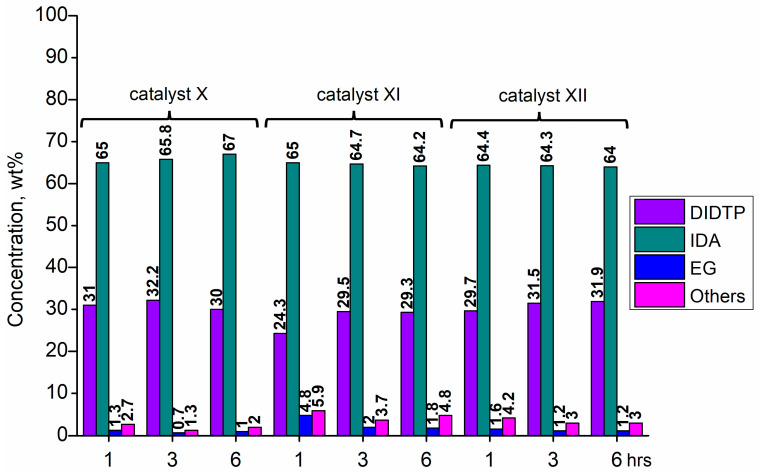
The reaction mixture composition during the depolymerization of PET waste using: dioctyltin oxide (**X**), cobalt (II) 2-ethylhexanoate (**XI**), and manganese (II) 2-ethylhexanoate (**XII**).

**Figure 6:**
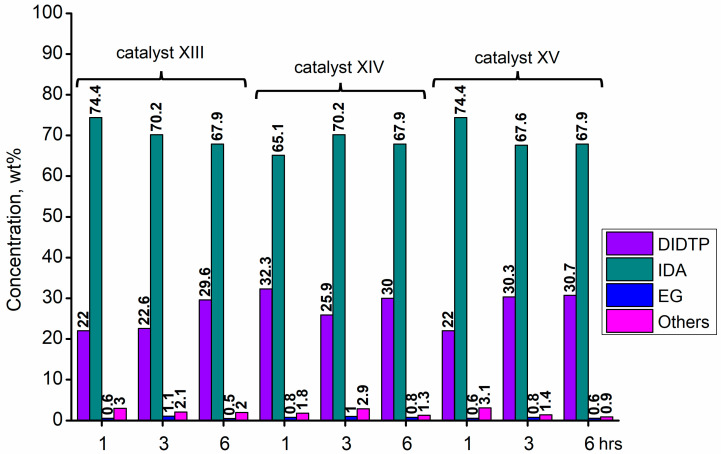
The reaction mixture composition during the depolymerization of PET waste using: 2-ethylhexanoate (**XIII**), zinc (II), 2-ethylhexanoate (**XIV**), and cobalt (II) neodecanate (**XV**).

**Figure 7:**
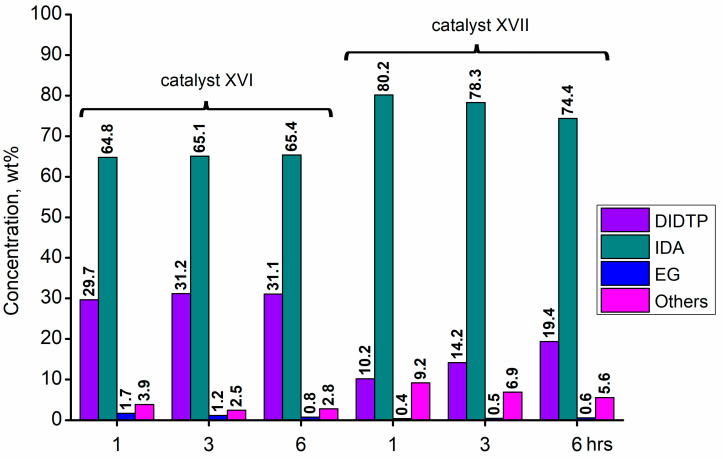
The reaction mixture composition during the depolymerization of PET waste using: zinc (II) neodecanate (**XVI**) and calcium (II) 3,5,5-trimethylhexanoate (**XVII**).

**Figure 8:**
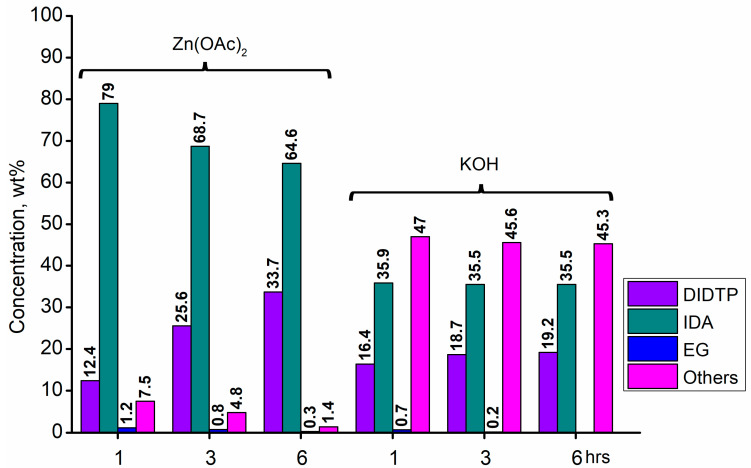
The reaction mixture composition during the depolymerization of PET waste using: zinc acetate and potassium hydroxide.

**Figure 9:**
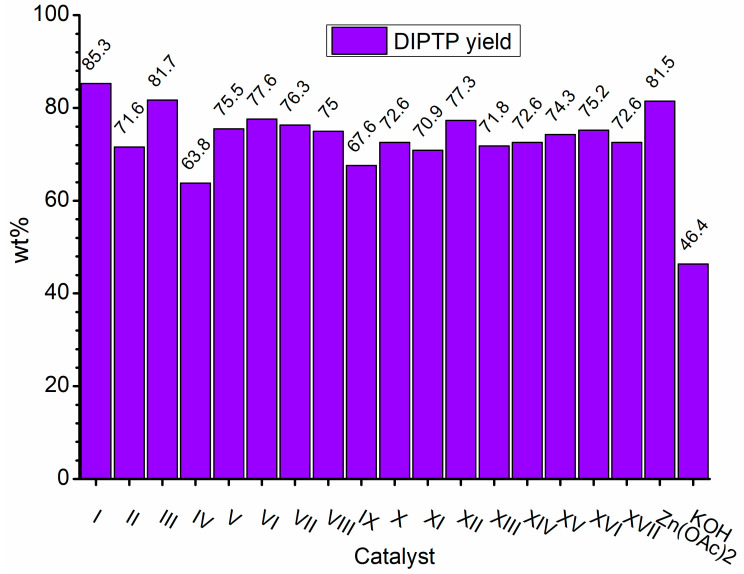
Yields of DIDTP obtained during catalyst screening.

Considering the results obtained for metallo-organic catalysts, a good catalytic activity in the alcoholysis of poly(ethylene terephthalate) (Figure 2, Figure 3, Figure 4, Figure 5, Figure 6, Figure 7, Figure 8 and Figure 9) was observed. Trials conducted using isodecyl alcohol resulted in complete PET degradation when the Lewis acid catalyst was applied. The post-reaction mixtures contained high amounts of diisodecyl terephthalate and varying amounts of byproducts. The critical factor affecting the speed at which PET is depolymerized is the size of PET particles. López et al. [82] detailed the correlation between the mean particle size and glycolysis of waste PET. They established a link between the particle size, PET conversion, and stirring rate, highlighting their significance in reactions between different phases. Le et al. [83] investigated changes in the morphology of PET particles during chemical depolymerization. The authors explored variations in the PET particle size and structures during glycolysis, both with and without a catalyst, as well as in the presence of a cosolvent. They concluded that the mechanism of PET flakes’ degradation was similar under non-catalytic and catalytic conditions. Initially, there were no alterations on the surface of the PET flakes, but after 0.5 h, degradation was observed. Microscopic pores formed on the polymer surface, facilitating the penetration of reagents into the PET substrate. The swelling of PET was also noted due to substrate penetration into the polymer matrix. Similar results were found for the zinc acetate catalyst [83]. However, in the context of this study, screening tests of organotin catalysts revealed that the initial decomposition of poly(ethylene terephthalate) occurred rapidly, with all PET completely dissolved after one hour in the case of the most active catalysts. The application of other metallo-organic compounds with a lower catalytic activity resulted in a slower PET degradation after a longer time. Since PET flakes were of a similar size in all test trials, we can surmise that the activity of the tested compounds was a determining factor in the PET degradation rate.

Another limiting factor in PET’s alcoholysis is the catalyst’s and PET solubility in the alcohol used for the reaction. The low solubility of reaction mixture compounds may seriously lower the rate at which reaction products are formed. Casas et al. reported such an observation [84] in triglyceride methanolysis using various organotin catalysts: tin (II) chloride, tin (II) acetate, tin (II) 2-ethylhexanoate, and tin (II) stearate. The limiting factor of the reaction was the low solubility of the catalyst and methanol in triglycerides. The mutual solubility of the reactants and catalyst was observed only after the temperature was increased to 150 °C. Similar observations were made by Ferreira et al. [85] in their study of various organotin catalysts in the methanolysis of triglycerides. Regarding the results gathered during catalyst screening, we concluded that such a phenomenon occurred only when zinc acetate and potassium hydroxide were used as catalysts. In these cases, the concentration of DIDTP after one hour of the reaction was relatively low. As the reaction time progressed, the reaction conditions facilitated PET degradation, leading to the increased solubility of isodecyl alcohol. Regarding other catalysts, the low solubility of PET in the reaction mixture had a comparatively minor impact on the overall process. 

Organotin catalysts have shown a good overall activity in the depolymerization of poly(ethylene terephthalate), resulting in DIDTP yields well above 70%, except monobutyltin dihydroxychloride (**IV**) and dibutyltin bis(1-thioglyceride) (**IX**). In the case of these catalysts, the initial DIDTP concentration after one hour reached 19.6 and 15.2% wt., reaching 24.8 and 26.4%wt after three hours, respectively. After six hours, only a slight increase in the DIDTP concentration was observed. The degradation of PET was also slow, and in both cases, PET dissolved in the reaction mixture after over four hours. The reaction yields were lower than those in other organotin catalysts and reached 63.8% and 67.6%,, which shows that organometallic tin compounds containing additional heteroatoms tend to have a lower activity. 

Calcium 3,5,5-trimethylhexanoate (**VII**) allowed to obtain 28.6% wt. after one hour of the reaction and 29.8% wt. after three hours, reaching 31.5% wt. of DIDTP after six hours. PET decomposition was achieved after three hours of the process. 2-ethylhexanoic acid salts **(II, XI, XII, XIII, and XIV)** exhibited lower activity, resulting in lower DIDTP concentration and yields. However, the final DIDTP concentrations were similar for all of these catalysts, reaching about 30.0% wt. The lowest DIDTP concentration after one hour was observed for **XIII,** reaching 22.0% wt. In this case, unreacted PET was observed in the reactor after 3 h of the reaction. Similar times of PET degradation were observed for other 2-ethylhexanoic acid salts. In some cases, higher amounts of byproducts in the post-reaction mixtures were observed. Increased amounts of byproducts were also observed in reactions catalyzed by catalysts **XVI** and **XVII**. After six hours, the byproduct concentration reached 2.8% wt. (**XVI**) and 5.6% wt. (**XVII**). A complete PET degradation was observed after 4 h for **XVI** and 5 h for **XVII**. The concentration of byproducts is significant because its excessive amount can cause difficulties during the purification of the product, especially in the distillation stage, where the thermal decomposition of such compounds may occur, influencing the purity of the final product. Tin (II) oxalate exhibited the best catalytic activity out of all tested catalysts. After one hour, the complete dissolution of PET was observed, and the reaction mixture contained 32.3% wt. of DIDTP, which increased to 34.3 and 35.2% wt. after three and six hours of the reaction, respectively. Most notably, the concentration of byproducts was very low after three hours of the process, and it reached 0.6% wt. and 0.4% wt. after six hours.

**Figure 10:**
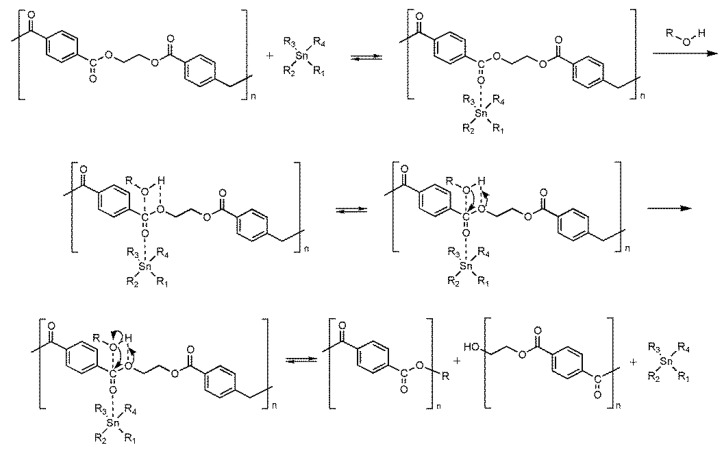
PET depolymerization mechanism catalyzed by the Lewis acid catalyst.

As PET alcoholysis essentially involves a transesterification process, it is reasonable to expect that the reaction mechanism is similar to the one proposed by Casas et al. [84]. This publication proposed the transesterification mechanism of vegetable oils catalyzed by the Lewis acid tin compound. The authors utilized tin acetate, tin chloride, tin 2-ethylhexanoate, and tin stearate in the methanolysis reaction. Figure 10 outlines a similar mechanism for PET alcoholysis. In the initial stages of the reaction, an oxygen atom from the carbonyl group bonded with the free tin orbitals, constituting Lewis acid sites and enhancing the electrophilicity of the carbonyl group carbon. Consequently, this increased the susceptibility to nucleophilic attack. Notably, the accessibility of the reaction center for the alcohol was crucial, especially for higher alcohols such as 2-ethylhexanol, isononyl alcohol, and isodecyl alcohol. 

Interestingly, the applied catalysts showed no signs of deactivation due to the use of multicolored waste PET. However, it was reported by Hofmann et al. [54] that the application of waste poly(ethylene terephthalate) can lead to a significant decrease in reaction yields. Different contaminants decreased significantly, from the reported 95% for colorless PET to 38% for green transparent PET. 

Considering the composition of the post-reaction mixture, mainly the DIDTP concentration and byproducts, tin (II) oxalate (**I**) was selected as the optimal catalytic compound for the depolymerization process. The good catalytic activity of catalyst (**I**) allowed to obtain a post-reaction mixture with a high DIDTP content and a low byproduct formation. The activity of the selected catalyst also was not hindered by the impurities that are present in waste feedstock, as was observed in other catalytic systems reported in the scientific literature [54].

### 2.2. Synthesis of Application Samples

**Figure 11:**
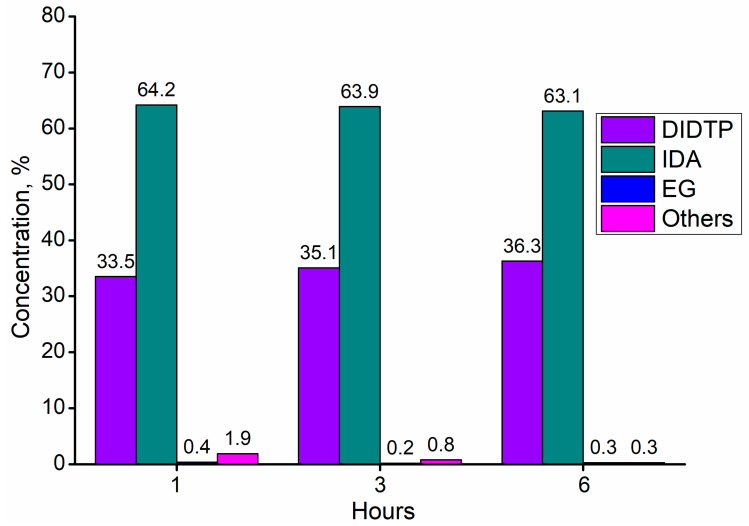
The composition of the post-reaction mixture obtained from application batch synthesis.

**Figure 12:**
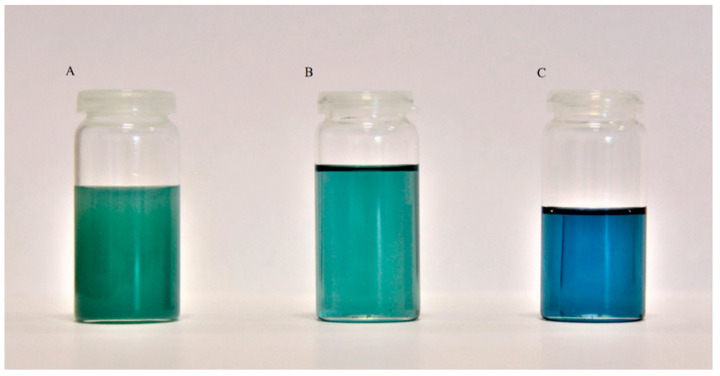
Samples obtained after 1 (**A**), 3 (**B**), and 6 h (**C**) of synthesis of the application batch.

The synthesis of the application samples was conducted as described in the Methods Section. Tin (II) oxalate was used as a catalyst due to the good yield of DIDTP and low concentration of undesired byproducts. Test samples were collected after 1, 3, and 6 h of the reaction (Figure 11). The results show that the concentration of DIDTP increased steadily during the depolymerization process, increasing from 33.5% wt. after 1 h to 63.1% wt. after 6 h. Simultaneously, the concentration of isodecyl alcohol decreased from 64.3% wt. after 1h to 63.1% wt. after 6 h of the reaction. The largest increase in the DIDTP concentration was observed during 1st hour of the process. This increase was due to the fact that, during the initial stage of depolymerization, PET undergoes degradation. At the same time, the polymer itself is penetrated by alcohol and reacts in the presence of a catalyst to form low-molecular PET chains, which further reacts with ID alcohol to form DIDTP. Furthermore, the partial degradation of PET resulted in increased polymer solubility in the reaction environment. This also contributed to an increase in DIDTP after 1 h of the process. Because the application process is conducted on a larger scale than screening trials, the amount of PET was significantly increased. This resulted in an increase in the overall surface area of PET that is available for the reaction with alcohol. Furthermore, PET depolymerization, which is a transesterification reaction, is an equilibrium process. In its initial stage, this reaction progresses fairly quickly due to the low concentration of alcoholysis products. Increasing the concentration of the main product causes a slower increase in its content in the post-reaction mixture. The concentration of DIDTP and ID alcohol changed only slightly after 1st hour of the reaction due to the large amount of alcohol used in this process, which also impacted the concentration of byproducts in the reaction mixture. The amount of byproducts decreased from 1.9% wt. after the 1st hour of the reaction to 0.8% wt. after the 3rd hour to 0.3% wt. after 6 h. The low byproduct content plays an important role in the further purification of the post-reaction mixture. The reaction progress can also be observed visually. During the 1st hour of the reaction, PET degrades almost completely, and only small amounts of fine sediment are present in the reactor. After three hours, the reaction mixture becomes transparent, indicating that all of the PET degraded, and most formed DIDTP (Figure 12). A gradual change in the color of the reaction mixture was observed. This can be attributed to pigment decomposition at higher temperatures combined with the effect of the catalyst and alcohol on the pigments present in the waste PET. The reaction mixture had a similar composition to that obtained during catalyst screening for tin (II) oxalate. The concentration of DIDTP was higher, while the byproduct content was marginally lower. The reaction yield was higher and reached 88% compared to the 85% obtained during catalyst screening. Taking into account these observations, we conclude that the reaction scale-up had a positive impact on the process of the depolymerization of waste poly(ethylene terephthalate). 

**Figure 13:**
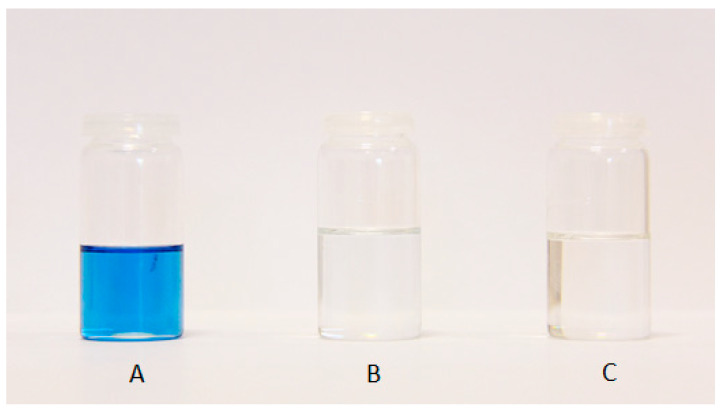
Samples of the purified and unpurified ester (**A**) after 2-ethylhexanol, (**B**) distilled ester, and (**C**) ester after further purification.

**Figure 14:**
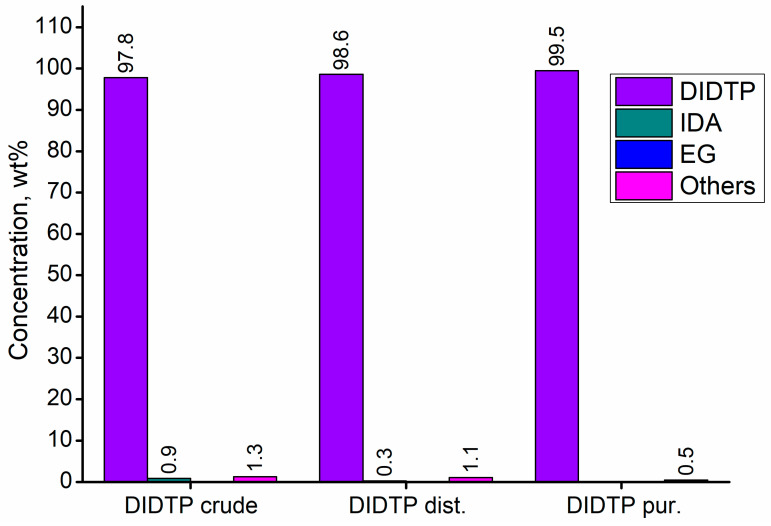
The composition of the obtained plasticizers at different stages of purification.

Following the parameters outlined in Table 1, the distillation of the post-reaction mixture yielded the results depicted in Figure 13. Initially, a heteroazeotrope of water and isodecyl alcohol was distilled. The water originated from the purification stage, where the catalyst was washed out from the post-reaction mixture with an alkali solution, followed by washing with distilled water to remove the applied hydroxide. After water removal, pure isodecyl alcohol was distilled, resulting in the crude product, a transparent blue viscous liquid (Figure 13). The distillation of isodecyl alcohol produced a crude terephthalate ester comprising 97.8%wt DIDTP and minor amounts of ID alcohol and 1.3% wt. other compounds. The further distillation of crude DIDTP followed the parameters outlined in Table 2, leading to a significant reduction in byproducts concentration. The refined product contained 98.6% wt. DIDTP, 0.3% wt. isodecyl alcohol, and 1.1% wt. of other compounds. The distillation of the terephthalate ester facilitated the separation of pigments from the ester, resulting in a clear, colorless, viscous liquid distillate (Figure 14). The further purification of the distillate with bleaching earth and activated charcoal yielded a pure product comprising 99.5% wt. DIDTP and 0.5% wt. other compounds. Consequently, the employed method for synthesizing DIDTP from PET waste, coupled with the purification technique, enabled the production of two types of plasticizers with varying levels of purity. The crude ester contained lower amounts of the desired ester and impurities such as pigments, while the high-purity product consisted of 99.5% wt. terephthalate ester. The plasticizing properties of these products were assessed accordingly.

### 2.3. GC-MS Analysis of Depolymerization Products

**Figure 15:**
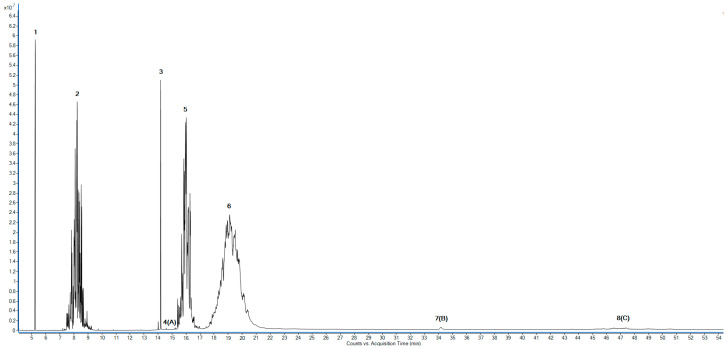
GC chromatogram of the post-reaction mixture obtained in the synthesis of the application sample.

The composition of the reaction mixture was determined using the GC_MS method. A sample obtained during waste poly(ethylene terephthalate) depolymerization was analyzed to determine its quantitative composition. The obtained chromatogram of the analyzed sample is presented in Figure 15.

The analysis revealed the presence of ten compounds in the post-reaction mixture (Table 1), among which monoethylene glycol (**1**) was a main byproduct of the alcoholysis of poly(ethylene terephthalate) and isodecyl alcohol (**2**) was used in excess to ensure a good PET conversion and low amount of byproducts. Terephthalic acid (**3**), a product of complete PET degradation (**2**), is present in the reaction mixture usually if the reaction is conducted in the presence of water and when PET hydrolysis occurs and if the applied catalyst favors hydrolysis reaction. Other identified compounds are intermediate products of poly(ethylene terephthalate) alcoholysis with isodecyl alcohol. The largest identified molecules (9, 8) represent dimers of terephthalic acid, where (9) is partially esterified by isodecyl alcohol. Molecules represented by these structures undergo further transesterification to form isodecyl-2-hydroxyethyl terephthalate (6) and bis(hydroxyethyl) terephthalate (4), which subsequently react with isodecyl alcohol to form DIDTP (**7**).

### 2.4. Physicochemical and Plasticization Properties of the Obtained Plasticizers

The purified ester’s physicochemical properties were determined and compared to the quality requirements of the commercially available DOTP (Oxoviflex®, Grupa Azoty Kedzierzyn S.A.) (Table 2).

As shown in Table 2, the physicochemical properties of the purified DIDTP were similar to those of the commercially available plasticizers. There is a slight difference in viscosity; however, the variance is negligible. This is because DIDTP has a different chemical structure than the commercial plasticizer (DOTP). Other parameters are within the required values. The distillation of the obtained crude plasticizer allowed to obtain a product with a low concentration of volatile substances and free acids. Most notably, the ester obtained using such a method meets the final product’s color requirements. The crude DIDTP also demonstrated physicochemical properties similar to those of commercial plasticizers; however, there are discrepancies in the dynamic viscosity, which is higher than required for commercial plasticizers. It is most likely caused by the presence of partially degraded PET present in the crude plasticizer.

**Figure 16:**
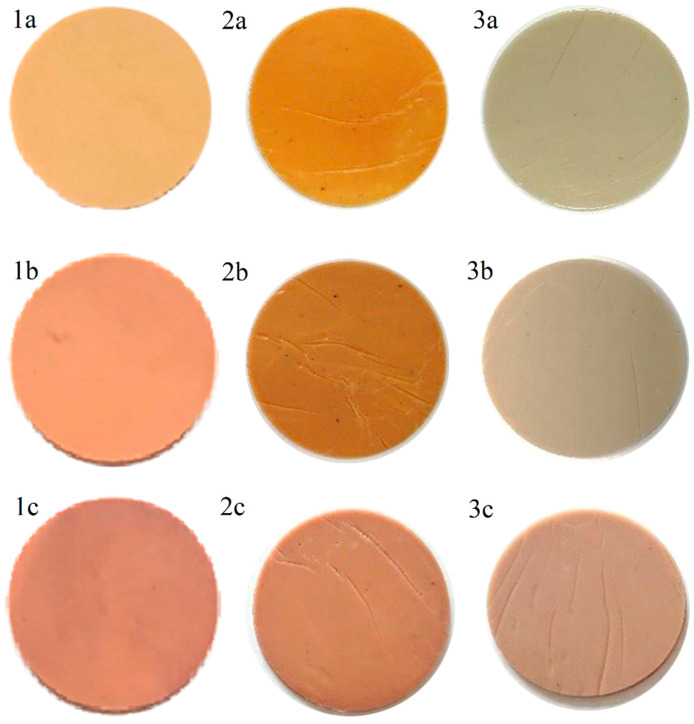
Samples of PVC samples containing plasticizers during the measurement of plasticizer migration. (**1**). Sample containing commercial plasticizer. (**2**). Sample containing distilled plasticizer. (**3**). Sample containing crude plasticizer. (**a**). Beginning of the measurement. (**b**). After 7 days. (**c**). After 28 days.

The plasticization properties were measured using crude and purified DIDTP and commercial dioctyl terephthalate and diisodecyl phthalate, which have similar plasticizing properties to DIDTP. Measurements were conducted according to the method described previously in Section 3 (Methods). The obtained results are summarized in Table 3. The mixture obtained from purified DIDTP had a similar hardness and density as the commercial plasticizer (DIDP). Tensile strength and elongation at break had slightly higher values compared to DIDP. However, plasticizer migration was at a similar level in both cases. Interestingly, crude DIDTP, when used in PVC mixtures, exhibited different properties than DIDP. Using crude DIDTP resulted in obtaining a mixture with a higher hardness of 95.4 ^o^ShA compared to 94.7 ^o^ShA and a higher density of 1.326 g/cm^3^ to 1.288 g/cm^3^ of DIDP. The application of crude DIDTP also resulted in a higher tensile strength of the PVC mixture of 25.6 Mpa compared to 20.4 Mpa for the commercial DIDP. The elongation at break was lower, reaching 220%, when in the case of a mixture with DIDP, it reached 250%. Most notably, plasticizer migration after 7 and 28 days was lower for crude DIDTP, reaching 13.6 and 21.5% wt., respectively, compared to 16.7 and 25.8% wt. for DIDP. These differences are caused by the presence of partially degraded PET, which results in the plasticizer exhibiting some of the properties of polymeric plasticizers. However, a low concentration of partially degraded PET causes only a slight reduction in plasticizer migration since the typical polymeric plasticizers tend to have a lower migration ratio compared to polymers. When comparing the obtained DIDTP plasticizer to the tested commercial DOTP mixtures, they have a higher hardness, tensile strength, and elongation at break and a lower plasticizer migration. Interestingly, plasticization using DOTP requires a longer time than in the cases of DIDTP and DIDP. PVC samples exhibited a change in color during plasticization measurements (Figure 16). The samples containing the commercial and purified plasticizers had a similar color initially; however, the samples obtained with the distilled plasticizer had a slightly darker color (Figure 16(1a,2a)). The color change was similar during migration measurement, shifting from light to dark brown. The samples obtained using crude DIDTP had a greenish color resulting from pigment presence. In this case, the color changed over time to gray after 7 days and later to light brown after 28 days. 

## 3. Materials and Methods

### 3.1. Materials

For this research, tin (II) oxalate (I) 95% wt., tin (II) bis(2-ethylhexanoate) (II) 97% wt., monobutyltin oxide (III) 98% wt., monobutyltin dihydroxychloride (IV) 96% wt., monobutyltin tris(2-ethylhexanoate) (V) 97% wt., dibutyltin diacetate (VI) 96% wt., dibutyltin oxide (VII) 97% wt., dibutyltin dilaurate (VIII) 98% wt., dibutyltin bis(1-thioglyceride) (IX) 97% wt., dioctyltin oxide (X) 97% wt., cobalt (II) 2-ethylhexanoate (XI) 90% wt., manganese (II) 2-ethylhexanoate (XII) 70% wt., zirconium (IV) 2-ethylhexanoate (XIII) 70% wt., zinc (II) 2-ethylhexanoate (XIV) 95% wt., cobalt (II) neodecanate (XV) 90% wt., zinc (II) neodecanate (XVI) 90% wt., calcium (II) 3,5,5-trimethylhexanoate (XVII), 90% wt. (Figure 1), potassium hydroxide 90% wt., and zinc acetate (>97% wt.) were used as catalysts. The tin catalysts were supplied by PMC Organometallix, Mount Laurel, NJ, USA, while other metallo-organic catalysts were supplied by Borchers Americas, Inc., Westlake, OH, USA Potassium hydroxide was supplied by Merck Sp. z o.o., Warsaw, Mazowieckie, Poland, and zinc acetate dihydrate by Chempur, Piekary Śląskie, Śląskie, Poland. Isodecyl alcohol (99.7% wt.) was purchased from XIAMEN AMITY INDUSTRY&TRADE Co., Ltd., Xiamen, China. Waste PET comprised >95% wt. of shredded consumer waste bottles with flakes sized >12 mm, supplied by PRT Radomsko Sp. Z o.o. Radomsko, Śląskie, Poland. The following auxiliary materials were used for the plasticizer purification: activated carbon PAC200C supplied by Norit and Rafinol 910FF supplied by Brenntag, Kędzierzyn Koźle, Opolskie, Poland. Plasticization trials were conducted with the application of polyvinyl chloride (Polanvil S-70) supplied by Anwil S.A. Włocławek, Kujawsko-Pomorskie, Poland, chalk Extra-1 supplied by ZSChiM “Piotrowice II”, Piotrowice, Świętokrzyskie, Poland, and thermal stabilizer Baeropan R 8890 KA/2 supplied by Baerlocher GmbH, Unterschleißheim, Germany. Gas chromatography (GC) analysis was carried out with the use of: ethylene glycol standard 99.95% wt. supplied by and, 2-ethylhexyl alcohol standard 99.78% wt., pyridine >99.5% wt. supplied by Merck Sp. z o.o., Warsaw, Mazowieckie, Poland, DOTP 97.7% wt. supplied by Acros Organics, Waltham, MA USA, N-methyl-N-trimethylsilyl-trifluoroacetamide (MSTFA) >99% wt supplied by Larodan, Solna, Sweden, and chloroform 98.5% wt supplied by POCh, Gliwice, Śląskie, Poland.

### 3.2. Methods

#### 3.2.1. Catalyst Screening Tests and Plasticizer Synthesis

An analog methodology to the one described by DuPont [64,65] and Muszyński [81] was executed for the depolymerization procedure. A Dean–Stark apparatus, a reflux condenser, a mechanical stirrer, a temperature regulation system, and an inert gas inlet were attached to a 250 cm^3^ reactor. A total of 75% volume of the Dean–Stark (DS) apparatus was filled with distilled water and the remaining space with isodecyl alcohol (ID). Distilled water was used to efficiently separate ethylene glycol (EG), a main byproduct generated during the reaction. Since the EG solubility is higher for water than for alcohol, and water with ID is immiscible, forming two layers, with alcohol on the top, it becomes possible to remove EG from the reaction mixture at an efficient rate. Alcohol in excess to PET of 4.6 was used to provide an efficient PET decomposition and to ensure a low concentration of reaction byproducts. In the reactor, 40 g of PET waste and 185 g of ID were loaded and heated until 180 °C. Once the desired temperature was attained, 0.3 g of the catalyst (constituting 0.75% wt. relative to PET) was introduced. The temperature was progressively elevated throughout the reaction to 230 °C to maintain a constant reflux through the DS apparatus. For each experiment, three samples were taken after 1, 3, and 6 h of the ongoing process. The product yield was calculated using the following formula:

YPET[%]=mDIDTPMDIDTPmDIDTPMDIDTP·100

where mDOTP is the mass of bis(isodecyl)terephthalate in the reaction mixture at a given time; MDOTP is the molar mass of bis(isodecyl)terephthalate (446.7 g/mol); mPET is the initial mass of poly(ethylene terephthalate); and MPET is the molar mass of PET repeating units (192 g/mol).

#### 3.2.2. Application Samples’ Synthesis

For the increased synthesis scale of diisodecyl terephthalate, a similar methodology was applied to the one described in the Catalyst screening tests and plasticizer synthesis Section. Into a 20 dm^3^ reactor, 2000 g of waste PET (comprising a mixture of colorless and colored variants) was loaded, along with 9250 g of ID and 15 g of catalyst (0.75% wt. of PET). The complete dissolution of PET was observed after 2h of the reaction. The reaction proceeded for a total duration of 6h, followed by a sample of the reaction mixture being taken to mark the acid value. 

The resulting post-reaction mixture was then cooled to 60 °C, and a solution containing 20% wt. of water with excess KOH was added to neutralize the acidic compounds present in the reactor. The quantity of the KOH was determined based on the acid value, with a 10% wt excess. The neutralization procedure was carried out for over 1 h, and then, the phase separation proceeded. The nonpolar phase was then washed three times with distilled water to eliminate any remaining KOH. Subsequently, the excess alcohol present in the mixture was distilled from the crude product under reduced pressure.

ID was removed from diisodecyl terephthalate (DIDTP) through distillation with the parameters depicted in Table 4. The resulting crude DIDTP was split into two fractions. The first fraction, containing pigments and minor quantities of denser unreacted compounds, was directly employed in plasticization experiments to assess the impact of impurities on the plasticizer characteristics. The remaining fraction underwent vacuum distillation to yield purified DIDTP. Distillation was conducted under the parameters presented in Table 5.

The distillate of DIDTP exhibited a faint yellow tint and, therefore, underwent further purification employing 0.5% wt. of bleaching earth (Rafinol 910FF) and 0.5% wt. of activated carbon (Norit PAC 200C) at 90 °C for a duration of 1 h. After this treatment, the mixture separated under vacuum, yielding a colorless, transparent, thick liquid product.

#### 3.2.3. Analysis of Depolymerization Reaction Composition

Samples from the depolymerization reaction mixture and purified DIDTP were examined utilizing a Perkin Elmer’s Autosystem XL gas chromatograph, featuring a flame ionization detector and a nonpolar chromatographic column ZB-5 with dimensions of 60 m × 0.32 mm × 1 μm. The measurement parameters were set as follows: an injection volume of 0.5 μL, a carrier gas flow of 1.5 cm^3^/min, and a split ratio of 70:1.

Before analysis, the samples, in the form of a sparingly soluble precipitate (0.5 g), underwent extraction with a mixture of pyridine and chloroform (1:1) using 1 cm^3^ of the solvent. The resulting suspension was then filtered using a syringe filter. Subsequently, 50 cm^3^ of the filtrate was combined with 0.1 cm^3^ of pyridine and 0.1 cm^3^ of MSTFA. After 30 min, 0.2 cm^3^ of chloroform was introduced into the mixture.

The calibration process was executed with the following protocol: A solution containing ID and DIDTP was prepared at a concentration of 200 mg/mL in pyridine. Aliquots of 100, 50, 12.5, and 5 μL were withdrawn from the solution and supplemented with pyridine to reach a total volume of 100 μL (equivalent to 20, 10, 2.5, and 1 mg of standards in 100 μL of pyridine). After adding 100 μL of MSTFA to each solution, they were further diluted with 200 μL with n-heptane and allowed to stand for 30 min at room temperature. The concentration of ID was determined based on its calibration curve, while the concentrations of other components were determined using the calibration curve of DIDTP.

The analysis was performed employing internal normalization, with the consideration of correction factors for the identified components. The concentrations of key molecules, including DIDTP, ethylene glycol, ID, and other compounds, were determined through this approach.

We measured the physicochemical properties of the obtained plasticizers.

The determination of the physicochemical properties was accomplished with the following methodologies:

Dynamic Viscosity:

Method: PN-EN ISO 3219:2000 “Plastics. Polymers/resins in the liquid state or as emulsions or dispersions. The viscosity was determined using a rotational viscometer at a specified shear rate”.

Equipment: spindle viscometer.

Temperature: 20 °C.

Kinematic Viscosity:

Method: PN-EN ISO 3104:2004 “Petroleum products. Transparent and opaque liquids. Determination of the kinematic viscosity and calculation of dynamic viscosity”.

Density:

Method: PN-EN ISO 3675:2004 “Crude oil and liquid petroleum products. Laboratory density determination. Method with hydrometer”.

Flashpoint:

Method: PN-EN ISO 2592:2017 “Determination of flash and burning temperatures. Cleveland’s open melting pot method”.

Volatile Compounds:

Method: PN–C-89401:1988 “Plasticizers—Test methods”.

Color Measurement:

Method: PN-81/C-04534.01 “Determination of the color of chemical products using the Hazen scale (platinum-cobalt scale)”.

#### 3.2.4. Plasticization Measurements

For the plasticization measurements, a mixture was formulated by combining PVC with the plasticizer (synthesized ester), stabilizer, and chalk. The ensuing physical properties of this mixture was then assessed.

Preparation of mixture with plasticizer: Plasticizer samples were evaluated in application tests to determine their plasticizing effects on PVC. PVC samples were blended using a rheomixer along with the plasticizer and commercially available DIDTP following the same method. The formulation for the mixtures consisted of 100 parts per hundred (PHR) of PVC, 50 PHR of plasticizer, 4.5 PHR of stabilizer, and 10 PHR of chalk.

The mixing process occurred in the preheated rheomixer at 85 °C for 5 min, after which the temperature was gradually increased to 115 °C, and mixing continued for an additional 5 min. Subsequently, the temperature was raised to 180 °C, facilitating the gelling of the mixture. Molds were prepared from the obtained samples, and fittings for individual tests were cut.

To ascertain the fundamental plasticizing properties, the following measurements were conducted with the PVC samples:

Hardness. Hardness was evaluated by applying a Zwick hardness tester following the PN-ISO 868 standard. The measurement procedure involved placing a sample on the table of the hardness measuring apparatus. Subsequently, the needle was lowered, and the result was read from the electronic display. This operation was repeated five times at different locations within the sample. The final hardness represented the arithmetic mean of the measurements.

Density. Density measurements were conducted using the Mettler Toledo AG-204, following the PN-EN ISO 1183-1 standard. The measurements were accomplished with the following methodology: After the initial weighing, the sample was submerged in water on the weighbridge and weighed again. Upon obtaining approval, the balance displayed the density result. This procedure was repeated three times, and the final result represented the arithmetic mean of the measurements.

Tensile strength and elongation at break. The tensile strength and elongation at break were determined using an Instron 4466 tensile compression tester, based on the PN-EN ISO 527-1 and PN-EN ISO 527-2 standards. The tests were conducted under ambient temperature and humidity conditions. The measurement procedure for determining tensile strength and elongation at break involved placing a sample of well-specified dimensions in the jaws of the apparatus. The measurement was initiated, and the jaws stretched the sample until it reached the point of breakage. This process was repeated five times for each sample. The computer program calculated the final results.

Plasticization. The plasticization time was determined using an internally standardized methodology with the Haake Poly-Lab QC periodical mixer equipped with the PolySoft OS software v3.3.2 and a Reomix 600 chamber. The measurement procedure involved heating the chamber of the periodic mixer to 85 °C. Subsequently, all loose raw materials and plasticizers were introduced into the apparatus and an appropriate program was initiated for 30 min. Then, the content of the chamber was heated to 120 °C. Throughout the entire process, the rotation speed of the rotors remained constant at 28 rpm. The camera recorded the change in torque values over time, and the measurement was conducted for approximately 15 min. Based on the obtained results, graphs depicting the dependence of the torque value on time were generated. The time at which the plasticization of the mixture occurred was extrapolated from the graph.

The sample forming. The process involves creating samples by mixing the plasticizer with PVC and other compounds, utilizing the hydraulic press LP-S-50. The pressing procedure was as follows: A fitting containing the PVC sample was positioned between two metal plates protected with polyester film. The plates were inserted into the press, and the designated program was initiated. After cooling, the necessary fittings were cut for further analysis.

Plasticizer mass loss. The test was conducted by the PN-EN ISO 177:2003 standard. Three repetitions were performed for each sample, and the average value was determined. The measurement procedure involved the following steps: 

Discs from the obtained PVC molds and polyethylene (PE) without additives were cut with specific dimensions: PVC discs with a diameter of 50 ± 1 mm and a thickness of 1 ± 0.5 mm; PE discs with a diameter of 60 ± 1 mm and a thickness of 1.5 ± 0.5 mm.

Each disc was measured by weighing and measuring the thickness and diameter.

The PVC disc was placed between two PE absorption discs.

Each set of three discs was positioned into a column between two glass plates measuring 10 × 10 × 0.5 cm.

The columns were placed in a forced air dryer at 70 °C for 7 and 28 days under a load of 5 kg.

Three repetitions for each sample were performed.

The arithmetic mean from the obtained results for each sample was calculated.

## 4. Conclusions

This paper conducted screening trials of seventeen organometallic catalysts for PET depolymerization using isodecyl alcohol, and their activity was compared to that of two reference catalysts (zinc acetate and potassium hydroxide). A large-scale synthesis was conducted in the laboratory using the most efficient catalyst. Additionally, the physicochemical and plasticizing properties of the resulting plasticizers were evaluated. 

The research demonstrated that the organometallic compounds displayed a higher catalytic activity than the reference catalysts. They were effective in the PET chemical recycling process in the presence of isodecyl alcohol. The most active catalysts facilitated the degradation of the majority of PET within the first hour of the reaction. It was found that the organotin catalysts containing heteroatoms exhibit a lower catalytic activity than those without additional heteroatoms. The most efficient catalyst, tin (II) oxalate, yielded diisodecyl terephthalate at a concentration of 32.3% wt. within the first hour and 25.2% wt. after 6 h, yielding 81.67%. The concentration of other byproducts was very low, decreasing to 0.4% wt. by the end of the process. Overall, tin catalysts usually exhibited a higher catalytic activity than other tested organometallic catalysts, except organotin catalysts containing additional heteroatoms. 

Tin (II) oxalate was the most effective depolymerization catalyst in large-scale plasticizer synthesis. The scale-up of PET depolymerization allowed to obtain a post-reaction mixture with a higher DIDTP concentration than that during the catalyst screening process, revealing the positive influence of increasing the process scale on the reaction. Since the application process was conducted on a larger scale than the screening trials, the amount of PET was significantly increased. This resulted in an increase in the overall surface area of PET that is available for reaction with alcohol. The scaled-up alcoholysis of waste PET resulted in a post-reaction mixture containing 36.3% wt. of DIDTP, 63.1% wt. of isodecyl alcohol, 0.3% wt. of ethylene glycol, and 0.3% wt. of other byproducts.

The application sample produced two types of plasticizers: purified and unpurified. After purification, two products containing 97.8% wt. and 99.5% wt were obtained. The study revealed that their individual physicochemical and plasticizing properties were comparable to those of commercial plasticizers, except for the tensile strength, which was lower for commercial DIDP and DOTP (20.4, 25.6, and 21.1 MPa for commercial, unpurified, and purified DIDT, respectively).

To summarize, the catalysts chosen for this study, particularly tin (II) oxalate, can be effectively employed in the production of terephthalate plasticizers from PET waste. This will enable the utilization of new feedstock for terephthalate plasticizer production while simultaneously contributing to the reduction in unrecycled plastic. Furthermore, PET recycling methods will diminish the demand for new terephthalic acid, positively impacting the environment and decreasing the amount of PET waste.

## Figures and Tables

**Table 1:** Structures of the compounds identified in the post-reaction mixture using GC-MS.

Compound	Name and Structure of Identified Compound	Molar Mass [Da]
1.	Ethylene glycol	62
2.	2-propyl-1-pentanol	130
3.	2-etylhexanol	130
4.	2-ethylhexanoic acid	144
5.	Diethylene glycol	106
6.	Ethylene glycol mono(2-ethylhexanoate)	188
7.	2-Ethylhexyl 2-ethylhexanoate	256
8.	2-ethylhexyl benzoate	234
9.	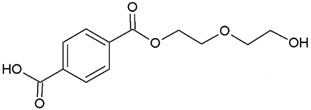 Mono[2-(2-hydroxyetoksy)ethyl] terephthalate	254
10.	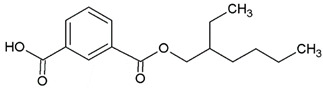 Mono(2-ethylhexyl) isophthalate	278
11.	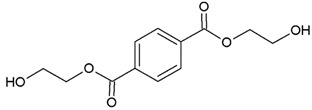 *Bis*(2-hydroxyethyl) terephthalate	254
12.	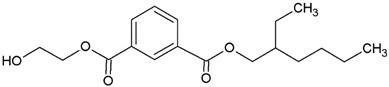 2-Ethylhexyl 2-hydroxyethyl isophthalate	322
13.	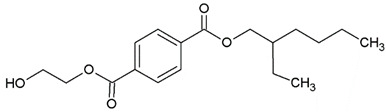 2-Ethylhexyl 2-hydroxyethyl terephthalate	322
14.	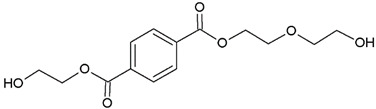 2-Hydroxyethyl[2-(2-hydroxyethoxy)ethyl] terephthalate	298
15.	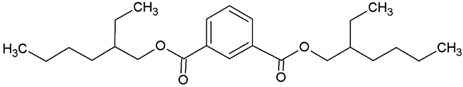 *Bis*(2-ethylhexyl) isophthalate	390
16.	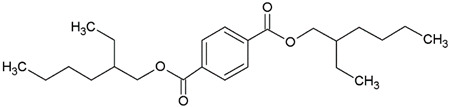 *Bis*(2-ethylhexyl) terephthalate	390
17.	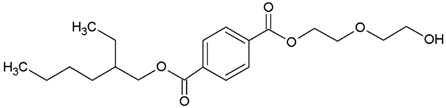 2-ethylhexyl[2-(2-hydroxyethoxy)ethyl] terephthalate	366
18.	Polymer fragments	>400

**Table 2:** The physicochemical properties of the purified DOTP and requirements for the commercial plasticizer.

Parameter	Crude DIDTP	Purified DIDTP	Commercial Plasticizer Parameters
Dynamic viscosity 25 °C [mPas]	85.97	63.81	65.8
Density 20 °C [g/cm^3^]	0.976	0.959	0.980–0.985
Flashpoint [°C]	246	225	min. 230
Volatile substances 150 °C [%wt.]	0.25	0.15	max 0.2
Free acids [%wt.]	0.1	0.08	max 0.1
Color [Haz]	-	5	>20

https://oxoplast.com/wp-content/uploads/2014/08/01_Oxoviflex_Specyfikacja_2020.11.02_PL.pdf (accessed on 26 November 2024).

**Table 3:** Plasticization properties of the synthesized (purified and unpurified) and commercial plasticizers in PVC mixtures.

Sample	Hardness [oShA]	Density [g/cm^3^]	TS [MPa]	Elongation at Break [%]	Plasticization Time [min]	Plasticizer Migration [%wt]	
						7 Days	28 Days
**DOTP (comm.)**	**90.0**	**1.298**	**18.5**	**230**	4.5	22.2	34.0
DIDP (comm.)	94.7	1.288	20.4	250	2	16.7	25.8
DIDTP (distilled)	94.6	1.287	21.1	260	1	16.9	25.7
DIDTP (crude)	95.40	1.326	25.6	220	2	13.6	21.5

TS—tensile strength.

**Table 4:** Isodecyl alcohol distillation parameters.

Process Stage	T_v_ [°C]	T_t_ [°C]	P [mbar]
Start	130	110	15
End	210	112	15

T_v_, temperature in distillation vessel; T_t_, temperature on top of the distillation column.

**Table 5:** DIDTP distillation parameters.

Process Stage	T_v_ [°C]	T_t_ [°C]	P [mbar]
Start	258	252	2.8
End	291	275	2.8

T_v_, temperature in distillation vessel; T_t_, temperature on top of the distillation column.

## Data Availability

The original contributions presented in the study are included in the article; further inquiries can be directed to the corresponding authors.
